# A brief history of organoids

**DOI:** 10.1152/ajpcell.00120.2020

**Published:** 2020-05-27

**Authors:** Claudia Corrò, Laura Novellasdemunt, Vivian S.W. Li

**Affiliations:** Stem Cell and Cancer Biology Laboratory, The Francis Crick Institute, London United Kingdom

**Keywords:** disease modeling, organoids, precision medicine, regenerative medicine

## Abstract

In vitro cell cultures are crucial research tools for modeling human development and diseases. Although the conventional monolayer cell cultures have been widely used in the past, the lack of tissue architecture and complexity of such model fails to inform the true biological processes in vivo. Recent advances in the organoid technology have revolutionized the in vitro culture tools for biomedical research by creating powerful three-dimensional (3D) models to recapitulate the cellular heterogeneity, structure, and functions of the primary tissues. Such organoid technology enables researchers to recreate human organs and diseases in a dish and thus holds great promises for many translational applications such as regenerative medicine, drug discovery, and precision medicine. In this review, we provide an overview of the organoid history and development. We discuss the strengths and limitations of organoids as well as their potential applications in the laboratory and the clinic.

## INTRODUCTION

The modern term organoid refers to cells growing in a defined three-dimensional (3D) environment in vitro to form mini-clusters of cells that self-organize and differentiate into functional cell types, recapitulating the structure and function of an organ in vivo (hence, also called “mini-organs”). Organoids can be derived from either embryonic stem cells (ESCs), induced pluripotent stem cells (iPSCs), or neonatal or adult stem cells (ASCs) (52, 70) through a process similar to the way in which the organ acquires its distinctive organization. Self-organization within the organoid occurs through spatially restricted lineage commitment and cell sorting, which requires activation of various signaling pathways mediated by intrinsic cellular components or extrinsic environments such as extracellular matrix (ECM) and media.

ASC-derived organoids are generated directly from postnatal or adult tissues either from single ASC or ASC-containing tissue units. This is supported by a cocktail of growth factors in the culture media that recapitulate signaling control under normal tissue homeostasis. Besides normal tissues, ASC-derived organoids can also be established from patient-specific material for disease modeling and precision medicine (see organoid applications below). On the other hand, ESC/iPSC-derived organoids involve stepwise differentiation protocols using various growth factors or inhibitors that resemble the developmental cues during gastrulation and organogenesis. The pluripotent property of ESCs and iPSCs enables the generation of organoids from all three germ layers. This is particularly useful for studies of early-stage embryonic development, where primary human material is limited. In this review, we will discuss the history and development of 3D organoid culture and provide the most recent update on organoid research that covers whole range of systems. We will explore various applications of organoid technology in biomedicine and discuss its promises and challenges. Finally, we will evaluate the pros and cons of 3D organoid technology compared with other conventional models.

## 3D CULTURE MODELS: FROM CELL AGGREGATES TO ORGANOIDS

The 3D culture system is established by suspension culture to avoid direct physical contact to the plastic dish. This can be achieved using scaffold or scaffold-free techniques. Scaffolds are biological or synthetic hydrogels that resemble the natural ECM. The most commonly used one is Matrigel, which is a heterogeneous and gelatinous protein mixture secreted by Engelbreth-Holm-Swarm (EHS) mouse sarcoma cells (99). It comprises mainly adhesive proteins such as collagen, entactin, laminin, and heparin sulfate proteoglycans, which resemble the extracellular environment to provide structural support and ECM signals to the cells. For scaffold-free techniques, cells are cultured in droplets of a defined culture medium hanging from a plate by gravity and surface tension (146). Alternatively, the 3D structure of the organoids can also be established via “air-liquid-interface.” In this case, cells are cultured on a basal layer of fibroblasts or Matrigel that are initially submerged in medium, which gradually evaporates and exposes the upper cell layers to the air to allow polarization and differentiation (61, 147).

Back in 1907, Henry Van Peters Wilson described the first attempt of in vitro organism regeneration, where he demonstrated that dissociated sponge cells can self-organize to regenerate a whole organism (159). A few decades later, several groups performed dissociation-reaggregation experiments to generate different types of organs from dissociated amphibian pronephros (46a) and chick embryos (158). In 1964, Malcolm Steinberg introduced the differential adhesion hypothesis, proposing that cell sorting and rearrangement can be explained by thermodynamics mediated by differential surface adhesion (135). Stem cell research began to thrive when pluripotent stem cells (PSCs) were first isolated and established from mouse embryos in 1981 (34, 84). But it was not until 1998 that scientists were able to isolate and culture embryonic stem cells derived from human blastocysts for the first time (145). Later on, iPSCs were subsequently established by the reprogramming of mouse and human fibroblasts, which has brought significant impact to stem cell and organoid research (139, 140, 164).

**Fig. 1.**
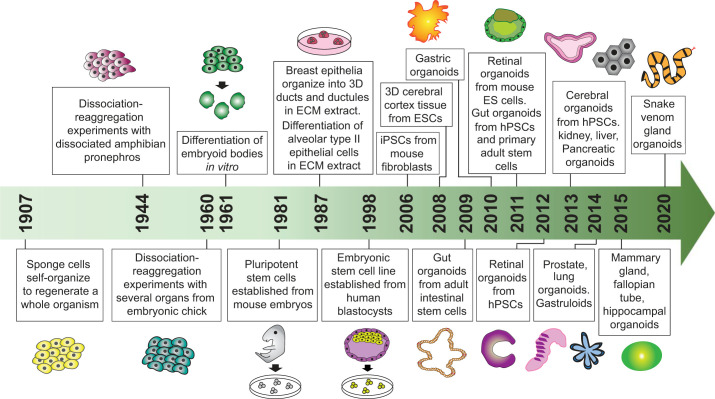
Timeline for the development of organoid cultures. A summary of key landmark studies and breakthroughs leading to the establishment of various organoid technologies. 3D, 3-dimensional; ECM, extracellular matrix; ESCs, embryonic stem (ES) cells; hPSCs, human pluripotent stem cells; iPSCs, induced pluripotent stem cells.

In 1987, scientists began to improve cell culture conditions by simulating the in vivo microenvironment. Li et al. (75) demonstrated that breast epithelia can form 3D ducts and lumen when grown on EHS ECM extract, where they appeared to be able to synthetize and secrete milk protein as opposed to two-dimensional (2D) culture. Similarly, alveolar type II epithelial cells were able to maintain their differentiation in the presence of ECM matrix (128), highlighting the importance of cell-matrix interactions in tissue maintenance and differentiation. Organoid research began to shift from 2D to 3D when Eiraku et al. (31) were able to generate cerebral cortex tissue from ESCs using the 3D aggregation culture method. In 2009, a landmark study from Sato et al. (119) showed that single leucine-rich repeat containing G protein-coupled receptor 5 (Lgr5)-expressing adult intestinal stem cells can form 3D intestinal organoids in Matrigel that self-organize and differentiate into crypt-villus structures in the absence of a mesenchymal niche. This was the first report on establishing 3D organoid culture derived from a single ASC, which set the scene for many subsequent organoid works in other systems, including mesendoderm (e.g., stomach, liver, pancreas, lung, and kidney) and neuroectoderm (brain and retina) using either ASCs or PSCs (Fig. 1). Below, we provide the most recent updates on organoid technology in various systems.

## PROGRESS IN ORGANOID RESEARCH

### Gastrointestinal Organoids

The gastrointestinal (GI) tract arises from the endoderm during development, which forms a tube that can be divided in three different regions: the foregut, the midgut, and the hindgut (17). The foregut gives rise to the oral cavity, pharynx, respiratory tract, pancreas, stomach, and the liver, the midgut gives rise to the small intestine and the ascending colon, and the hindgut gives rise to the remaining colon and the rectum. Understanding the molecular mechanism and signaling regulation underlying the GI tract development and homeostasis is crucial for establishment and maintenance of ASC/PSC-derived organoids from these regions.

### Intestinal Organoids

In adult intestine, Wnt and Egf are known to play key roles for stem cell maintenance in the crypt, whereas Bmp drives differentiation in the villi (76, 87). In 2009, Sato et al. (119) described the first establishment of long-term 3D culture of intestine organoids from single Lgr5+ stem cells. These organoids were grown in Matrigel in the presence of Wnt agonist R-spondin, Egf, and Bmp inhibitor Noggin to form crypt-villus structures and were able to differentiate into all intestinal cell types, recapitulating the organization and function of the small bowel in vivo. Similar protocols for long-term culture of human colon, adenoma, and adenocarcinoma were subsequently established (59, 118). Importantly, transplantation of these intestinal organoids in mice showed long-term engraftment into the damaged colonic epithelium in vivo, highlighting the regenerative potential of these 3D organoids (165). Building on these adult-derived organoid cultures, a modified protocol of human PSC-derived intestinal organoids was further established (133). In particular, human PSCs were first treated with activin A to drive mesendodermal identity, followed by Wnt3a and Fgf4 to promote hindgut specification. The hindgut spheroids were subsequently cultured in Matrigel following the adult-derived organoid protocol to promote maturation. The major difference of PSC-derived intestinal organoids from adult-derived ones is the presence of surrounding mesenchymal cells in the culture, which allows formation of both epithelium and mesenchyme supported by mouse vasculature upon engraftment in vivo (157).

### Gastric Organoids

Stomach and intestinal epithelia share many molecular and physiological similarities, including the presence of proliferating Lgr5+ stem cells at the base of the glands/crypts. With minor modification of the intestinal culture system, gastric organoids were established from adult mouse pyloric Lgr5+ stem cells or Troy+ chief cells in corpus gland with the addition of Wnt3a and Fgf10 (6, 134). A similar method was adopted for the establishment of long-term culture of human adult gastric organoids (7). Subsequently, human PSC-derived gastric organoids were generated by adding Wnt3a, Fgf4, Noggin, and retinoic acid (RA) to drive posterior foregut fate, followed by 3D culture in Matrigel for maturation (86). These PSC-derived organoids are believed to adopt predominantly pyloric lineage.

### Tongue and Salivary Gland Organoids

Apart from intestine and stomach, organoids derived from tongue in the upper GI tract have also been explored. The initial approach was to derive lingual organoids from Bmi-expressing stem cells from adult tongue epithelium, which formed stratified squamous epithelia without salivary acinar cells or taste bud cells (46). Later on, taste bud organoids were established using LGR5+, LGR6+, or CD44+ stem/progenitor cells derived from taste buds in circumvallate papilla tissue with taste receptor expression (3, 109). Moreover, long-term expansion of mouse salivary gland organoids driven by Wnt signals has also been reported (83). More recently, it has been shown that transcription factors Sox9 and Foxc1 can drive differentiation of mouse ESC-derived oral ectoderm to salivary gland organoids, which can mature to functional salary gland following orthotopic transplantation (144).

### Liver and Pancreatic Organoids

The liver derives mainly from the foregut endoderm epithelium during development that gives rise to the hepatic bud structure, which generates hepatoblasts and subsequently hepatocytes and biliary epithelium (167). An early study showed that dissociated chick embryonic hepatic tissue can reaggregate and form secretory units with functional bile ducts (158). Adult liver and pancreas are slow cycling under homeostasis. It has been shown that cycling Lgr5+ cells were found near the bile ducts after damage in mice (50). These cells were able to generate organoids (budding cysts) when grown in 3D culture conditions with Matrigel and can be differentiated to form mature, functional hepatocytes (50). These liver organoids consist mostly of progenitor cells expressing bile duct and hepatocyte markers but can differentiate into functional hepatocytes when transplanted into a mouse model of liver disease (50). In a follow-up study, long-term expansion of adult bile duct-derived bipotent progenitors was established from human liver (51). In 2018, two studies further reported the successful long-term expansion of human and mouse hepatocyte as 3D organoid culture with high engraftment efficiency (47, 103). An alternative method has also been described to generate vascularized human liver from human iPSCs (143). This protocol involves differentiation of human PSCs into hepatic endodermal cells in 2D together with human mesenchymal stem cells and human endothelial cells. When grown in Matrigel, these cells spontaneously form vascularized 3D aggregates that can further engraft in vivo to form functional liver with the vascular network.

Pancreatic organoids can also be generated by plating mouse embryonic pancreatic progenitor cells in Matrigel (42). Similarly, mouse and human pancreatic organoids were subsequently established from adult pancreas, which can further differentiate to ductal and endocrine lineages after transplantation (14, 49).

### Brain Organoids

Vertebrate central nervous system is derived from the neuroectoderm during development (106). The human brain is a highly complex system that can be broadly divided into three regions, forebrain, midbrain, and hindbrain, that are composed primarily of neurons and glia cells. Previous dissociation-reaggregation experiments using chick neural progenitors derived from early developing brain formed clusters of neuroepithelial cells in a radial manner around a lumen similar to the neural tube, suggesting a self-organizing capacity of these brain cells (54). Similarly, neural progenitor cells (NPCs) also have the ability to aggregate and form neurospheres in suspension culture with the capacity to differentiate into neurons and astrocytes (110). Neural aggregates can also be generated from PSC-derived embryoid body (EB) (168). More recently, neural rosettes were further established from PSCs, which contained NPCs surrounding a central lumen resembling the neural tube (32). Remarkably, they can be further specified into various mature cell types with characteristics of different brain regions (35, 66, 79, 80, 125, 163). However, these models are still largely based on 2D culture or simple aggregates, which lack the complexity for the study of brain development and function.

Watanabe and colleagues (155, 156) have pioneered in developing 3D culture of different brain regions from mouse or human PSCs to recapitulate the complex brain tissue organization. They first generated forebrain tissues by plating mouse (155) or human (156) EBs in 2D. When transferred to 3D aggregation culture, these neuroepithelium formed more complex structures recapitulating the dorsal forebrain (31). This 3D protocol was further optimized later on to allow self-organization of neuronal layers similar to early cortical development that can be cultured up to 112 days (60). Different brain regional identities can also be developed from ESCs by manipulating growth factors such as Hedgehog, Fgf, Bmp, and Wnt (23, 89, 136).

In 2013, Lancaster et al. (71) further established the 3D cerebral organoids that contain different brain regions within single organoids. This is an improved method from Watanabe and colleagues (155, 156) by embedding EBs in Matrigel, which allows polarization and outgrowth of large neuroepithelial buds. These mini-brains can further grow up to a few millimeters when transferred to spinning bioreactor and develop into different brain regions, including retina, dorsal cortex, ventral forebrain, midbrain-hindbrain boundary, choroid plexus, and hippocampus. Subsequent studies further generated other organoid protocols to model specific brain regions, such as midbrain-specific organoids (58), hippocampal organoids (115), and cerebellar organoids (88). Using 3D printing technology, a miniaturized spinning bioreactor was further generated to allow cost-effective generation of forebrain-specific organoids from human iPSCs (107).

### Retinal Organoids

The neuroectoderm-derived retina originates from optic vesicle during development, where the front of the vesicle invaginates to form two adjacent epithelial layers: the outer retinal pigmented epithelium and the inner neural retina (44). Reaggregation experiments in chick retina showed self-organization of retina in vitro (87a, 134a). These reaggregates can further organize into a correctly laminated structure when cultured in the presence of Wnt2b (91). Later on, 3D culture of mouse EB aggregates further allowed the establishment of optic cup organoids resembling early retina with retinal stratification and apical-basal polarity (30). Optic cup organoids can also be generated from human PSCs (92). These human retinal organoids are larger than mouse organoids and have the capacity to grow into multilayered tissue containing both rods and cones.

### Kidney Organoids

The kidney arises from the intermediate mesoderm through Wnt and Fgf signaling, which develops into the ureteric bud and the metanephric mesenchyme to form early renal tubes (82). Similar to other tissues, dissociation-reaggregation experiments in chick and mouse embryonic kidney demonstrated the ability to self-organize and form organotypic renal structures (148, 158). In 2013, ureteric bud organoids were established from human PSCs that were first cultured in Bmp4 and Fgf2 for mesodermal specification, followed by exposure to RA, activin A, and Bmp2 to generate ureteric bud-committed renal progenitors (160). These human progenitor cells were further cocultured with disaggregated mouse embryonic kidney cells to self-organize and form 3D ureteric bud structures. In addition, metanephric mesenchyme identity can also be generated from mouse EB and human PSCs by sequential exposure to activin, Bmp4, and the Wnt activator CHIR99021, followed by RA and Fgf9 (138). Coculture of the metanephric mesenchyme with spinal cord tissue forms 3D structures with organized nephric tubules and glomeruli. Similarly, hESCs can also be differentiated to ureteric and metanephric progenitors through primitive streak and intermediate mesoderm, which further form 3D structures similar to ureteric epithelium and proximal tubules when cocultured with dissociated mouse embryonic kidney (141). In 2015, a simplified and improved protocol was established by direct differentiation of human PSCs to complex multicellular kidney organoids that contain nephrons associated with a collecting duct network surrounded by endothelial cells and renal interstitium (142). More recently, long-term culture of kidney tubular organoids was further established from adult human or mouse kidney tissues or from human urine, which form proximal and distal nephron segments (120).

### Other Organoid Types

Organoids can be generated from a broad range of tissues in addition to the ones mentioned above. For example, Jamieson et al. (57) have recently established mammary organoids from single adult mammary epithelial cells containing polarized secretory epithelium surrounded by myoepithelial cells. Prostate organoids can also be derived from adult mouse and human prostate epithelia to form both luminal and basal cells (19, 62). Thyroid organoids were generated by transient expression of the transcription factors NKX2–1 and PAX8 to direct mouse ESC differentiation into thyroid follicular cells and form 3D follicular structures when treated with thyrotropin (4). Cardiovascular organoids can be generated from EBs by modulating substrate stiffness (129). Lung organoids can be generated by coculturing adult bronchioalveolar stem cells and lung endothelial cells in Matrigel (72). Similarly, human airway organoids were established from bronchoalveolar resections that comprise basal cells, functional ciliated cells, mucus-producing cells, and CC10-secreting club cells (114). Stable fallopian tube organoids were also established from human fallopian tubes containing both ciliated and secretory cells (63). In addition, pituitary organoids have also been generated from EBs when grown under ectoderm-promoting conditions, which can further mature and synthesize pituitary hormones (137). A similar protocol has been used to generate inner ear organoids from EBs, which consist of functional inner ear sensory epithelia with stereocilia and kinocilia (68).

Besides modeling individual organs, organoids have also been recently used to explore early mammalian embryonic development. Embryonic organoids or gastruloids were established by 3D aggregation of mouse ESCs in suspension that developed into embryo-like structures with polarized gene expression in the absence of external asymmetry clues (152). These embryonic organoids self-organize and exhibit behaviors reminiscent of mammalian gastrulation, giving rise to cell types that correspond to the three germ layers with axial organization in a time scale similar to mouse embryos. Comparison of mouse gastruloids and embryos further reveals somitogenesis dynamics, highlighting the power of these gastruloids as a model for exploring early embryonic development in vitro (151). It will be important to further develop equivalent gastruloid systems in primates to model human embryo development in vitro.

In fact, organoid technology has also been extended to other animal models in addition to mouse and human. Methods of generating intestinal, mammary, keratinocyte, and liver organoids have been reported in different species such as bovine, porcine, ovine, chicken, feline, and canine (5, 29). A recent study has further reported the generation of snake venom gland organoids that express high levels of toxin transcripts, which can potentially be used for toxicology studies (105).

Despite the diversity of organoid systems and their corresponding culture protocols, there are some core growth factors and chemical modulators shared between systems. In particular, vast majority of the ASC-derived organoids are cultured in Matrigel suspension, which requires serum-free basal media supplemented with Wnt agonists and/or ligands (R-spondin, Wnt3a), Egf, and BMP inhibitor (Noggin). Depending on the signaling or hormonal requirements of their tissues of origin, additional growth factors or inhibitors [such as FGF in liver (47, 50, 51), gastric (6, 7, 134) and pancreatic (14, 49) organoids, gastrin in gastric organoids (6, 7, 134), and dihydrotestosterone in prostate organoids (19, 62)] are added to the cultures. On the other hand, stepwise differentiation protocols are required for ESC/PSC-derived organoids. Notably, activin A is required to drive differentiation of ESCs/PSCs to definitive endoderm, whereas Fgf and Wnt can promote neuromesoderm differentiation (21, 41). In essence, the similarities and differences of the culture protocols between systems reflect the growth signal requirement during development and tissue homeostasis.

## ORGANOID APPLICATIONS

**Fig. 2.**
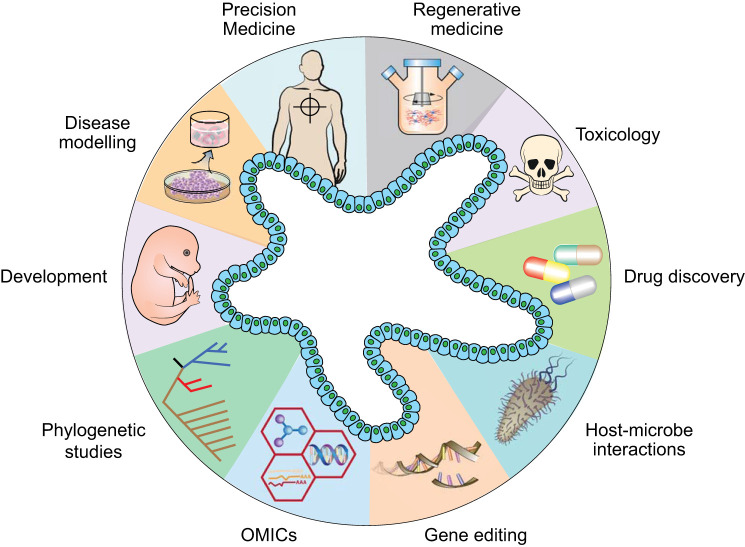
Diverse applications of organoid technology. Schematic diagram summarizing various applications of organoids in many areas, including developmental biology, disease modeling, precision medicine, regenerative medicine, toxicology, drug discovery studies, host-microbiome interactions, gene editing, multiomics, and phylogenetic studies.

Organoids are becoming one of the mainstream cell culture tools in many biomedical studies. The wide range of tissue types, the long-term expansion capacity, and the physiological 3D architecture of organoids make them a powerful new technology for many biological and clinical applications. Notably, organoids have been widely used for development and disease modeling, precision medicine, toxicology studies, and regenerative medicine (Fig. 2). Below, we focus on the applications of organoids in disease modeling, biobanking, precision medicine, and regenerative medicine.

### Disease Modeling

#### Genetic diseases.

Cystic fibrosis (CF) is an autosomal recessive genetic disease caused by mutations in the cystic fibrosis transmembrane conductance regulator (CFTR) chloride channel. In 2013, Dekkers et al. (24) generated the first human CF-patient derived intestinal organoids carrying F508del *CFTR* mutation to recapitulate the disease in vitro. They developed a swelling assay where healthy organoids respond to Forskolin treatment by rapid swelling, whereas such an effect is strongly reduced in CF organoids. This organoid swelling assay has proven to be very reliable to predict responders to CFTR modulators and has become the first organoid-based personalized medicine application for CF patients in The Netherlands (8). Interestingly, gene editing by CRISPR-mediated homologous recombination in primary patient-derived organoids (PDOs) can repair the *CFTR* mutation and function, implying the potential application of such a gene correction approach to single-gene hereditary defects (121). Hereditary multiple intestinal atresia (HMIA) is another autosomal recessive disorder characterized by bowel obstructions. Pathogenic mutations in the tetratricopeptide repeat domain 7A (TTC7A) have been identified (117). Patient-derived intestinal organoids showed activation of the RhoA kinase pathway and apicobasal polarity inversion, which could be restored by adding RhoA kinase inhibitor (Y-27632) (13). Similarly, liver organoids derived from patients with α1-antitrypsin (A1AT) deficiency and Alagille syndrome can also recapitulate the in vivo pathology, where accumulation of misfolded precipitates of A1AT protein in hepatocytes and biliary defects were observed respectively (51).

Cerebral organoids have been used to model human microcephaly, a genetic disease caused by a mutation in *CDK5RAP2*, where organoids generated from patient-derived iPSCs were smaller with reduced progenitor regions (71). Forebrain organoids have been used to model a genetic condition that causes lissencephaly (smooth brain), which showed defects in progenitors and Wnt signaling (9, 53). Brain organoids could also be relevant models for neurodegenerative diseases such as Alzheimer’s disease (AD), the most common type of dementia, characterized by extracellular deposition of misfolded amyloid-β (Aβ)-containing plaques and intracellular neurofibrillary tangles (NFTs) (43, 153). Raja et al. (108) have developed a scaffold-free culture method to generate iPSC-derived brain organoids from patients with familial AD, which could reproduce several AD pathologies, like Aβ aggregation, hyperphosphorylated tau protein, and abnormalities of endosomes. Treatment of these patient organoids with β- and γ-secretase inhibitors can significantly reduce the Aβ and tau pathology, demonstrating the potential of using human brain organoids for drug discovery in AD (108). More recently, mini-brains have further been used to model Parkinson’s disease (PD) (132). These organoids were generated from midbrain floor plate NPCs containing midbrain dopaminergic neurons (mDANs) that resemble key features of the human midbrain to produce and secrete dopamine. PD PDOs carrying *LRRK2*-G2019S mutation recapitulated main features of the disease with decreased number and complexity of mDANs. In parallel, Kim et al. (65) have generated isogenic midbrain hiPSCs-derived organoids by introducing heterozygous *LRKK2*-G2019S point mutation using the CRISRP/Cas9 system to model PD. Transcriptome analysis of control versus mutant organoids identified thioredoxin-interacting protein as the key factor to mediate the *LRRK2*-G2019S pathological phenotype (65).

In addition, iPSC-derived retinal organoids carrying a mutation in *CEP290* have been used to model Leber congenital amaurosis, a ciliopathy that leads to inherited blindness. By restoring the expression of full-length CEP290, cilia length and protein trafficking in cilium were restored (100). Human PSC-derived lung bud organoids have also been used to model intractable pulmonary fibrosis by introducing mutation in *HPS1*, leading to accumulation of ECM and mesenchymal cells reminiscent of the features of fibrotic lung disease (18). Together, these results highlight the advantage of the 3D organoid-based culture system for studying genetic diseases.

#### Infectious diseases.

The 3D organoid technology offers excellent models for the study of host-pathogen interaction in different human infectious diseases involving viruses, bacteria, and protozoan parasites. For instance, cerebral organoids have recently been adopted to study the mechanisms of microcephaly caused by Zika virus infection that showed overall smaller sizes of infected organoids compared with controls, which is consistent with the pathology observed in patients (20, 40, 107). Treatment strategies have further been explored in these Zika-infected organoids to prevent the effects of Zika virus infection on neural progenitors (161, 170). Intestinal organoids also present valuable models to study a number of infectious diseases. For instance, by using human primary intestinal organoids, scientists suggested that human intestinal tract may serve as an alternative infection route for Middle East respiratory syndrome coronavirus (MERS-CoV), which has caused a major human respiratory infection outbreak in 2012 (169). Human enteroids (organoids derived from small intestine) have also been used to study norovirus, where nitazoxanide treatment showed great inhibition of norovirus replication through activation of cellular antiviral response, indicating the therapeutic potential (33). Other viral infection studies using organoid systems include rotavirus and enteric adenovirus using intestinal organoids, herpes simplex virus 1 and cytomegalovirus in cerebral organoids, and BK virus infection in human kidney organoids (29).

Organoids are also increasingly popular for modeling parasitic infections. In 2018, Heo et al. (45) showed that microinjection of *Cryptosporidium parvum* into human intestinal and lung organoids allows the parasites to propagate within the organoids and complete its complex life cycle, which was not possible previously in conventional 2D culture systems. Similarly, *Toxoplasma gondii* has been shown to successfully infect and propagate in bovine and porcine small intestinal organoids (25).

3D organoid constructs have also been employed to investigate the relationship between infectious pathogens and corresponding cancers. For instance, epidemiological association between *Helicobacter pylori* and stomach cancers has been investigated through coculture of the pathogen with gastric organoids (7). Similarly, fallopian tube organoids were used to model the long-term impact of *Chlamydia trachomatis* infections in the human epithelium that may contribute to the development of ovarian cancer (64). Other uses of intestinal organoids to model bacterial pathogenesis include *Escherichia coli*, *Vibrio cholerae*, *Clostridium difficile*, and *Shigella* (29). Very recently, primary human intestinal organoids have been used to study the genotoxic *pks+ E. coli* carrying the colibactin-producing *pks* pathogenicity island (104). Long-term exposure of the *pks+ E. coli* induces a distinct mutational signature that is absent from organoids exposed to the isogenic *pks*-mutant bacteria. Importantly, the same mutational signature is detected in a subset of colorectal cancer (CRC), implying that exposure to *pks+ E. coli* may be the direct cause of the mutational signature.

#### Cancers.

For many years, immortalized human cancer-derived cell lines were the fundamental in vitro models for cancer studies. Patient-derived xenografts (PDXs) have subsequently been developed to better model tumor tissue architecture and heterogeneity in vivo. Despite being physiological, PDXs are very costly and time-consuming. The emergence of organoid technology in recent years has opened up an unprecedented approach to model human cancers in vitro. Organoids derived from different mouse or human tumors have now been widely adopted for the study of different types of cancer. CRC organoids were first established from different anatomic sites and displayed distinctive sensitivities to Wnt3a and R-spondin (118). Human liver cancer organoids were derived from patients by extensive refinement of medium conditions to expand the three common subtypes: hepatocellular carcinoma, cholangiocarcinoma, and combined hepatocellular-cholangiocarcinoma (16). Long-term maintenance and enrichment of pancreatic ductal adenocarcinoma (PDAC) organoids have also been established from mouse and human primary tissues that retain the histoarchitecture and phenotypic heterogeneity of the primary tumors (14, 48, 127). In addition, primary breast cancer organoids have been reported to faithfully recapitulate the corresponding parent tumors in morphology, histopathology, hormone receptor status, and mutational landscape (112). Organoids of other cancer types have also been subsequently established, including gastric (78, 90, 93, 126), prostate (39), ovarian (69), brain (22), bladder (74), kidney (15), lung (114), and esophageal cancers (77).

Alternatively, human cancer can also be engineered by introducing pathological mutations to wild-type organoids, using gene editing tools such as gene transfer, CRISPR-Cas9, or RNA interference methods. For instance, colorectal adenoma-carcinoma sequence can be recreated by introducing driver mutations (*APC*, *KRAS*, *TP53*, *SMAD4*, and *PIK3CA*) to healthy wild-type organoids and form invasive carcinoma after transplantation (28, 85). Further interrogation of different *APC* truncating mutations in intestinal organoids revealed the critical regulatory region for pathological Wnt activation in CRCs (96). Similarly, Seino et al. (127) modeled PDAC organoids by engineering driver genes *KRAS*, *CDKN2A*, *SMAD4*, and *TP53* via CRISPR-targeting, which revealed an unexpected Wnt niche adaptive response mediated by *TP53* mutations.

Unlike 2D cancer cell lines, cancer-derived organoids often retain their tumor heterogeneity and are thus ideal for study of tumor evolution. By comparing organoids derived from primary colorectal tumors and metastatic lesions isolated from the same patients, Fujii et al. (38) revealed that these tumors shared the same common origin and driver mutations, implying that the driver mutations precede metastatic dissemination. Later on, Roerink et al. (111) generated clonal organoids derived from multiple single cells from three CRCs as well as from adjacent normal intestinal crypts to study intratumor diversification. Global mutational landscape was used to construct phylogenetic trees, which showed extensive mutational diversification in CRC cells and that most mutations were acquired during the final dominant clonal expansion of the cancer. Taken together, these 3D organoids present revolutionary in vitro tools for disease modeling, phylogenetic, and drug discovery studies.

### Biobanking and Precision Medicine

The long-term expansion capacity of organoids has opened possibilities for biobanking of disease-derived organoids. These biobanks represent valuable resources for clinical applications such as omics analysis for cancer stratification and drug screening for precision medicine. In the past few years, extensive efforts have been made to establish living organoid biobanks derived from many different tumor types, including colorectal (38, 150), gastric (162), liver (16, 97), pancreatic (27), breast (112), prostate (11), lung (81, 114), glioblastoma (56), and bladder (74) cancer. Large-scale genomic and functional analysis from various studies have shown that tumor-derived organoids can faithfully recapitulate the phenotypic and genomic features of the primary tumors both in vitro and in vivo after transplantation (14, 39, 73, 112, 150). Importantly, the tumor heterogeneity and clonal dynamics were preserved after serial passaging of PDOs, indicating that these “mini-tumors” are genetically stable with enormous clinical applicability (15). With increasing interest in the use of organoids for disease modeling, biobanking can soon be extended beyond cancer, such as intestinal and lung organoids for cystic fibrosis patients and liver organoids for patients with various metabolic diseases.

The PDOs also provide unique opportunities for precision medicine through drug screening and drug safety test. Failure of many drug developments in clinical trials could partly be attributed to the inadequate evaluation of the drug toxicity at the preclinical trial stage. The emerging 3D organoid technology with the ability to grow matched normal and tumor PDOs enables proper assessment of drug toxicity and the possibility to determine the optimal and effective doses that would kill tumor cells with minimal damage to normal tissue. For instance, liver and kidney organoids would be excellent platforms to evaluate potential drug-related hepatic and nephron toxicity.

Another important clinical application of PDOs is to screen for drug responders. In a recent study, a living PDO biobank has been established from patients with metastatic, colorectal, and gastresophageal cancer, with the aim to screen for a library of 55 drugs either in phase 1 to 3 clinical trials or in clinical practice such as epidermal growth factor receptor (EGFR), BRAF, and phosphatidylinositol 3-kinase (PI3K)/mammalian target of rapamycin (mTOR) inhibitors (154). The results showed that PDOs can faithfully recapitulate drug responses and predict clinical outcomes in patients. Another study has generated more than 100 primary and metastatic breast cancer PDOs for high-throughput screening of drugs targeting HER signaling that showed high correlation with clinical drug responses (112). Similarly, Broutier et al. (16) performed a compound screening on PDOs from hepatocellular carcinoma and identified ERK signaling as a potential therapeutic target for primary liver cancer. In addition, PDOs have also been used to screen for CFTR modulators (8), drug combination strategies (102), chemotherapy, and radiotherapy responses (98, 101). These findings provide supportive evidence that PDOs are powerful, unprecedented tools for disease modeling and drug screening, paving the way toward precision medicine.

### Regenerative Medicine

Currently, organ replacement therapy of diseased or damaged tissues relies largely on allogeneic transplantation. However, the shortage of matched donor tissues and complications of life-long immunosuppression represent some of the major challenges of organ transplantation. The recent organoid technology with high expansion capacity and genetically stable property suggests that PDOs could potentially be explored as alternative treatment strategies to organ transplantation. Following the first establishment of mouse intestinal organoids, Yui et al. (165) have demonstrated that mouse colonic organoids could indeed be expanded and engrafted into damaged mouse colon and formed functional crypt units. Similar results were observed using fetal progenitor-derived small intestinal organoids (37). Human PSC-derived intestinal organoids have also been subsequently transplanted to mice under kidney capsule and showed crypt-villus structure with permeability and peptide uptake functions, highlighting the translational potential for treatment of short bowel syndrome and other gastrointestinal diseases (157). Organoids can also be combined with synthetic or biological (decellularized) scaffolds to engineer intestinal grafts in vitro (36, 67, 123).

Besides intestinal organoids, mouse adult liver organoids have also been shown to rescue liver failure and prolong the survival rate after transplantation into fumarylacetoacetate hydrolase mutant mice, a mouse model for tyrosinemia type I liver disease, or chemically damaged liver (47, 50). Similarly, PSC-derived liver organoids were able to rescue acute liver failure and restore the hepatic functions (95). Treatment of common bile duct disorders has further been explored by engineering extrahepatic biliary tree using extrahepatic cholangiocyte organoids (116). The resulting engineered ducts could reconstruct the gallbladder wall and repair the biliary epithelium following transplantation.

In addition, organoids could potentially be combined with gene correction as an alternative approach to treat single-gene hereditary degenerative diseases. For instance, as proof of concept, gene correction of *CFTR* mutation in PDOs using CRISPR/Cas9 gene editing could repair the CFTR function (121). It will be important to explore the therapeutic potential of other single-gene-associated degenerative diseases, such as PD mediated by *LRRK2-G2019S* mutation, using gene corrected-PDOs.

Although the potential of organoid applications in precision medicine and regenerative medicine is promising and exciting, it is important to address the safety, ethical, and legal concerns before moving to the clinic. One of the major concerns is the informed consent and ownership of the PDOs and the associated commercial interests. It will be important to define how and to what extent organoids are related to donors and the subsequent governance of any organoid-associated data, such as all the omics data generated from PDOs. When considering the application of organoids in regenerative medicine, it is particularly important to address all of the safety and ethical concerns before applying to patients. For instance, a global regulatory consensus of stem cell products and therapies may be needed to resolve the discrepancies of the medical regulations between countries. Open dialogues between scientists, policy-makers, and the public are also needed to decide to what extent these technologies should be used in the clinic.

## STRENGTHS AND LIMITATIONS OF 3D ORGANOID CULTURES OVER CONVENTIONAL MODELS

Conventional 2D cell or tissue cultures have long been used to model human development and diseases. Despite being widely adopted in many biomedical studies, 2D cell lines are generally considered as nonphysiological, as they are mostly immortalized and lack tissue architecture and complexity. On the other hand, genetically engineered mouse models (GEMMs) and PDXs are considered to be improved in vivo alternatives to model biological processes of diseases. Although GEMMs are the current workhorses in developmental and cancer research, the production of GEMMs (from design to generation and breeding) often takes years to establish. In addition, GEMMs cannot 100% recapitulate human conditions (e.g., microbiome and diversity), genetics, and/or physiology, which may impact their predictive power in assessing clinical outcomes. PDXs are another step forward to model human cancer by xenotransplantation of patient material into immunodeficient mice, but the establishment of PDXs is inefficient and time consuming. The newly emerged ex vivo PDOs offer superior alternatives to cell lines, GEMMs, and PDXs for disease modeling. Below, we discuss the advantages and limitations of the 3D PDOs as compared with other disease models (Table 1).

**Table 1.** Comparison of different in vitro and in vivo disease models

Characteristic	2D Cell Lines	3D PDOs	PDXs	GEMMs
Establishment efficiency	Inefficient	Easy	Inefficient	NA
Maintenance time	Low	Moderate	Moderate to high	High
Reproducibility	High	Medium	Medium	High
Cost	Low	Moderate to high	High	High
Tissue organization	2D constrains morphogenesis	Self-organized in 3D resembling in vivo architecture	Conserved; recapitulate patient’s tissue	Conserved but murine specific
Heterogeneity	Homogenous	Heterogeneous	Heterogeneous	Heterogeneous
Cell function	Limited	Moderate	Conserved and relevant to human biology	Not always relevant to human biology
Stromal microenvironment	Absent	Mostly absent	Preserved, except for immune cell populations	Preserved
Functional analysis	Easy	Easy, but could be complicated by the presence of matrix	Easy after tissue sampling, complex in vivo analysis	Easy after tissue sampling, complex in vivo analysis
Disease modeling	Poor	Good	Good	Mediocre; possible, but challenging for some human diseases
Scalability	Easy	Limited to diffusion of nutrients	Limited to engraftment efficiency	NA
High-throughput assay	Easy	More difficult but possible	Difficult	Difficult
Drug screening	Not very physiological	More relevant to the patient	More relevant to the patient	Not always relevant to human diseases
Personalized medicine	Not possible	Possible	Possible	Not always possible

2D, 2-dimensional; 3D, 3-dimensional; NA, not applicable.

Generation of PDOs are relatively easy once the culture condition is optimized and can be derived from limited primary tissue materials such as needle biopsies, urine (120), or bronchial lavage material (114). On the contrary, derivation of cell lines from primary tissues is often inefficient and involves extensive adaptation to the 2D culture conditions, resulting in substantial genetic changes. Compared with immortalized cell lines, organoids are considered superior in recapitulating the 3D architecture, heterogeneity, and cell functions of the primary tissues and hence, are more physiologically relevant for modeling human diseases and predicting drug response. Other models such as PDXs and GEMMs can better recapitulate human diseases in vivo, yet they are very costly and labor- and time-consuming, and therefore, they are not suitable for high-throughput screening.

Although organoid technology bridges the gap between cell lines and in vivo models, there are still limitations of the current system. Despites being heterogeneous, most PDOs lack surrounding stromal cells in the culture, which fail to reconstitute the tumor microenvironment (TME). The TME includes not only the surrounding fibroblasts and endothelial cells but also immune cells and ECM. Lack of TME in PDOs may perhaps compromise the application to predict clinical outcome. For instance, the response rate to immunotherapy (such as checkpoint blockade) varies among tumor types despite being promising in the clinic. A potential in vitro screening platform will be important to predict the immunotherapy drug response for personalized medicine. However, most PDOs from solid tumors lack TME and are thus not suitable for such screening. A recent study reported the generation of PDOs from different cancer types using an air-liquid interface method that retains fibroblasts and immune cells in the culture, which could potentially be used for personalized immunotherapy testing (94). However, the fibroblasts and immune cells of these PDOs progressively decline over a 1- to 2-mo period, indicating that they can be used only for short-term disease modeling. Additionally, organoids generated from chordoma patients have also been shown to contain both PD-L1-positive tumor cells and PD-1/CD8-positive lymphocytes, and they displayed a marked response to nivolumab treatment (124). On the other hand, cocultures of PDOs and peripheral blood lymphocytes have also been explored to assess the efficiency of T cell-mediated killing of matched tumor organoids, whereas the coculture efficiency beyond 3 days has not been tested (26). These studies show that, with further optimization, PDOs may have the potential for immuno-oncology investigations.

Most organoids are suspended in Matrigel and cultured in media saturated with growth factors. The presence of Matrigel could affect functional/biochemical assays and complicate the cell harvesting and passaging as compared with 2D cell line culture. Also, the enriched growth factors surrounding the organoids may compromise the natural morphogen gradients of the tissues. Spinning bioreactors optimized for brain organoid culture may resolve some of these issues. However, Bhaduri et al. (12) have recently shown that cortical organoids ectopically activate cellular stress pathways that impair cell-type specification and thus do not recapitulate distinct cellular subtype identities or appropriate progenitor maturation. The data suggest that the fidelity of these mini-brain organoids requires further evaluation.

Apart from the limitations described above, there are still some practicality issues that need to be addressed before large-scale rollout to the clinic. For example, the high reagent cost for PDO production makes it unlikely to be affordable by patients or the healthcare system. Notably, scaling up of PDOs is not as easy as it is in cell lines due to the complex 3D culture system. Finally, developing consistent and standardized drug screening strategies and readout is critical to reliably predict the patient treatment outcome in the clinic.

## PERSPECTIVES

Ever since the report of the first long-term expansion of ASC-derived organoids in 2009 (119), it has become clear that organoid technology has unique and powerful properties to revolutionize the conventional in vitro research tools for modeling human development and diseases. In particular, organoid studies have bridged the longstanding gaps in developmental biology and precision medicine. The 3D architecture and heterogenous properties of organoids enable us to study cell lineage specifications with spatial and temporal information. The ESC/iPSC-derived organoids have opened up the possibilities for gastrulation studies and regeneration of patient-derived organs, which were largely limited by the use of disorganized EB previously. Importantly, the establishment of PDOs from various disease models has further bridged the studies between basic research and precision medicine by providing more efficient, physiological, and reliable models as compared with PDXs and 2D cell lines. Increasing evidence suggests that PDOs functionally recapitulate primary human cancers, which present valuable translational tools for disease modeling, biobanking, drug discovery, and precision medicine. However, there is still room for improvement in the current organoid culture. More effort will be needed to standardize the culture protocol and to monitor the tumor heterogeneity after prolonged culture, which can directly affect the drug screening results. It will also be important to develop an improved long-term expansion protocol, including the surrounding TME, to better recapitulate the primary tumors. In addition, organoids can also be combined with other recent bioengineering tools such as organ-on-a-chip for microfluidic studies (131). For instance, microfluidic devices have been used to investigate the behavior of immune cells toward tumor cells (1). Several studies have further developed multi-organoid approaches to model the kinetics of metastasis and drug responses (10, 130). Further research on the combination of organoid and engineering technologies will open up exciting avenues for the next-generation organoid platforms to model more complex human physiology and pathology as well as to exploit their potential in regenerative medicine.

## GRANTS

The authors’ research is supported by the European Union’s Horizon 2020 research and innovation program (668294) and the Francis Crick Institute, which receives its core funding from Cancer Research UK (FC001105), the UK Medical Research Council (FC001105), and the Wellcome Trust (FC001105).

## DISCLOSURES

No conflicts of interest, financial or otherwise, are declared by the authors.

## AUTHOR CONTRIBUTIONS

C.C., L.N., and V.S.W.L. prepared figures; C.C., L.N., and V.S.W.L. drafted manuscript; C.C., L.N., and V.S.W.L. edited and revised manuscript; C.C. and V.S.W.L. approved final version of manuscript.
